# New Insights in the Pathogenesis and Therapy of Cold Agglutinin-Mediated Autoimmune Hemolytic Anemia

**DOI:** 10.3389/fimmu.2020.00590

**Published:** 2020-04-07

**Authors:** Sigbjørn Berentsen

**Affiliations:** Department of Research and Innovation, Haugesund Hospital, Haugesund, Norway

**Keywords:** autoimmune hemolytic anemia, cold agglutinin disease, lymphoproliferative, complement, inflammation, therapy

## Abstract

Autoimmune hemolytic anemias mediated by cold agglutinins can be divided into cold agglutinin disease (CAD), which is a well-defined clinicopathologic entity and a clonal lymphoproliferative disorder, and secondary cold agglutinin syndrome (CAS), in which a similar picture of cold-hemolytic anemia occurs secondary to another distinct clinical disease. Thus, the pathogenesis in CAD is quite different from that of polyclonal autoimmune diseases such as warm-antibody AIHA. In both CAD and CAS, hemolysis is mediated by the classical complement pathway and therefore can result in generation of anaphylotoxins, such as complement split product 3a (C3a) and, to some extent, C5a. On the other hand, infection and inflammation can act as triggers and drivers of hemolysis, exemplified by exacerbation of CAD in situations with acute phase reaction and the role of specific infections (particularly *Mycoplasma pneumoniae* and Epstein-Barr virus) as causes of CAS. In this review, the putative mechanisms behind these phenomena will be explained along with other recent achievements in the understanding of pathogenesis in these disorders. Therapeutic approaches have been directed against the clonal lymphoproliferation in CAD or the underlying disease in CAS. Currently, novel targeted treatments, in particular complement-directed therapies, are also being rapidly developed and will be reviewed.

## Introduction

Cold-antibody autoimmune hemolytic anemias (cAIHAs) are mediated by autoantibodies characterized by a temperature optimum of the antigen-antibody (AgAb) reaction at 0-4^o^C. These hemolytic disorders account for ~25–30% of autoimmune hemolytic anemias (AIHAs) (1). Table 1 shows a further classification of cAIHAs (1–7). Cold agglutinins (CAs) are cold-reactive antibodies that are able to agglutinate red blood cells (RBCs) (8–10). Only CA-mediated AIHAs, i.e., cold agglutinin disease (CAD) and cold agglutinin syndrome (CAS), will be further addressed in this review. Although the term CAD, as coined by Schubothe in 1952 (11), originally included both these concepts, CAD should be distinguished from CAS (2, 3).

**Table 1:** Cold-antibody mediated autoimmune hemolytic anemias.

**Entity**	**Etiology**	**Autoantibody properties**	**Ig class**	**Complement activation**	**Predominant type of hemolysis**	**Incidence**
Cold agglutinin disease (CAD)	Primary (low-grade LPD)	Cold agglutinins, anti-I (rarely anti-Pr or anti-IH)	IgM	Classical pathway ++, terminal pathway (+)	Extravascular	Uncommon, mainly elderly people
Cold agglutinin syndrome (CAS)	Secondary (Mycoplasma, EBV; aggressive lymphoma)	Cold agglutinins, anti-I or anti-i (rarely anti-IH?)	IgM or IgG	Classical pathway ++, terminal pathway (+)	Extravascular	Rare, any age
Paroxysmal cold hemoglobin-uria (PCH)	Children: Mostly postviral. Adults: Tertiary syphilis or hematologic malignancy.	Non-agglutinating biphasic Ab, anti-P	IgG	Classical pathway +++, terminal pathway +++	Intravascular	Rare in children, ultra-rare in adults

*Ab, antibody; EBV, Epstein-Barr virus; Ig, immunoglobulin; LPD, lymphoproliferative disorder*.

*Based on data from Sokol et al. (1), Berentsen and Tjønnfjord (2), Jäger et al. (3), Barcellini et al. (4), Randen et al. (5), and Shanbhag and Spivak (6)*.

According to the recent international AIHA consensus document (3), CAD is defined as “an AIHA characterized by a monospecific direct antiglobulin test (DAT) strongly positive for complement fragment C3d and a cold agglutinin (CA) titer of 64 or higher at 4°C (3, 12–14). DAT for IgG is usually negative, but can be weakly positive in up to 20% of the patients (15, 16). There may be occasional cases with CA titer <64 (3, 16).” By definition, “patients may have a B-cell clonal lymphoproliferative disorder (LPD) detectable in blood or marrow but no clinical or radiological evidence of malignancy” (3), and there is evidence that CAD is a clonal LPD of the bone marrow in most, probably all cases (5, 17–19). This distinct clinicopathological entity should be called a disease, not syndrome (3, 20). These patients are obviously identical with those previously described as having “idiopathic” or primary CAD (21, 22). Table 2 summarizes the diagnostic criteria for CAD.

**Table 2:** Diagnostic criteria for cold agglutinin disease.

**Level**	**Criteria**	**Procedures, comments and reminders**
Required for diagnosis	Chronic hemolysis	As assessed by hemoglobin levels and biochemical markers of hemolysis
	Polyspecific DAT positive	Performed in most laboratories but insufficient for diagnosis
	Monospecific DAT strongly positive for C3d	DAT is usually negative for IgG, but occasionally weakly positive
	CA titer > 64 at 4^**o**^C	Blood specimen must be kept at 37-38^**o**^C from sampling until serum/plasma has been removed from the clot/cells
	No overt malignant disease or relevant infection	Clinical assessment for malignancy. Radiology as required. Exclusion of recent infection with *Mycoplasma* or EBV
Confirmatory but not required for diagnosis	Monoclonal IgMκ in serum (or, rarely, IgG or λ phenotype)	Blood specimen must be kept at 37-38^**o**^C from sampling until serum/plasma has been removed from the clot/cells
	Ratio between κ and λ positive B-cells > 3.5 (or, rarely, <0.9)	Flow cytometry in bone marrow aspirate
	‘CA-associated lymphoproliferative disorder' by histology	Bone marrow biopsy

*CA, cold agglutinin; DAT, direct antiglobulin test; EBV, Epstein-Barr virus; Ig, immunoglobulin. Previously published by Berentsen (23), reused under general permission, slightly modified. Copyright: The American Society of Hematology*.

CAS is a similar clinical-hematological syndrome further defined by “the presence of an associated clinical disease, for example infection, autoimmune disorder, overt evidence of a lymphoma (clinical or radiological), or other malignancy” (3, 20). Typical underlying infections are *Mycoplasma pneumoniae* pneumonia, Epstein Barr virus (EBV) infection, or, rarely, other specific infections (2, 24, 25).

## Immune Pathogenesis in CAD and CAS

### Origin of Cold Agglutinins

Like other IgM, CAs are produced by B-cells, predominantly at the lymphoplasmacytic cell stage (5, 26). However, IgM can also be produced by a smaller compartment of plasma cells that are long-lived and not targeted by chemoimmunotherapy (26, 27). The CA-producing cells are monoclonal in CAD as well as in CAS secondary to lymphoma, but polyclonal in CAS secondary to infection (2, 9).

The *IGVH4-34* gene, originally named V_H_4-21, is located on the q arm of chromosome 14. In CAD, this gene encodes for the CA IgM heavy chain in more than 85% (9, 28, 29). In contrast, the monoclonal IgM heavy chain molecule found in Waldenström macroglobulinemia (WM) is usually encoded by the *IGHV3* gene (30). Framework region 1 (FR1) of the heavy gene variable region is essential for recognition of the I antigen (31, 32); however, the affinity and specificity for I antigen binding also depends on the heavy chain complementarity determining region 3 (CDR3) and the light chain variable region (29, 33). Recent data suggest that “subtle differences in light chain multiple binding sequences may contribute to differences in thermal amplitude and clinical phenotype” (29).

The first cytogenetic change observed in CAD was complete or partial trisomy 3 (34). A recent study found trisomy 3 (+3 or +3q) in all of 12 samples from CAD patients who participated in a clinical trial. Nine of these had an additional trisomy 12 or 18, but never both (19). Malecka et al. also found that the Ig light chain gene *IGKV3-20* and, to lesser extent, the similar *IGHV3-15* gene are used in most patients (74%) and might contribute to the I antigen binding. The *IGKV3-20* CDR3 region is highly homologous in a subgroup of patients and correlated with younger age at diagnosis (29). This finding is consistent with specific antigen selection in this group of patients. Table 3 summarizes the heavy and light chain gene usage. Next generation sequencing together with flow cytometry-assisted cell sorting of bone marrow from 16 patients enabled Malecka and coworkers to identify recurrent mutations of *KMT2D* (69%) and *CARD11* (31%) (36, 37). In diffuse large B-cell lymphoma, *CARD11* mutations have been shown to induce constitutive activation of the NF-κB pathway (29, 38).

**Table 3:** Immunoglobulin heavy and light chain V gene usage in cold agglutinin disease and Waldenström macroglobulinemia.

**Gene**	**Cold agglutinin disease**	**Waldenström macroglobulinemia/lymphoplasmacytic lymphoma**
Heavy chain gene	*IGHV4-34* (>85%) (5, 9, 28, 29)	*IGHV3* (83%) (*IGHV3-23*, 24%) (5, 30)
Light chain gene	*IGKV3-20* (59%) (29, 35)	Not determined

Evidence of a clonal lymphoproliferative disorder (LPD) of the bone marrow has been recognized for decades (21). This LPD was previously perceived as being heterogeneous and was classified into several entities of low-grade LPD, frequently interpreted as lymphoplasmacytic lymphoma (LPL) or marginal zone lymphoma (MZL) (16, 18, 21, 39). A comprehensive histopathology study of 54 patients with CAD, however, showed a surprisingly homogenous type of lymphoid infiltration that has been termed “CAD-associated LPD” (5, 40).

**Figure 1:**
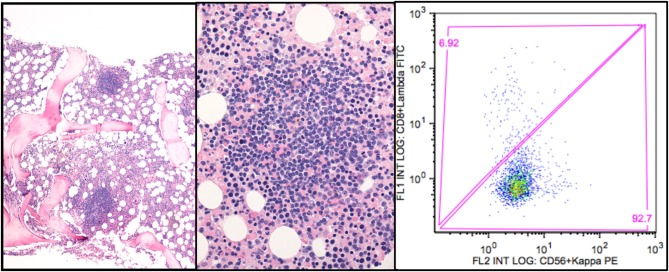
CAD-associated lymphoproliferative disorder. **(A)** shows the nodular infiltration pattern. **(B)** highlights the resemblance to marginal zone B cell infiltration. **(C)** shows the typical flow cytometry finding of a monoclonal κ+ B-cell population (gated on CD19+ B-cells). Courtesy of U. Randen. First published in Clin Adv Hematol Oncol 2020 by S. Berentsen et al. (41), reused under Creative Commons Attribution Non-Commercial License. Copyright: S. Berentsen, A. Malecka, U. Randen, and G.E. Tjønnfjord.

The lymphoid infiltration usually consists of nodular B-cell aggregates, but some biopsies show only scattered B-cells (Figure 1). Involvement can vary between 5 and 80% of the intertrabecular surface, median 10% (5, 41). Mature plasma cells are seen surrounding the lymphoid nodular aggregates and throughout the marrow in between, but only few plasma cells are usually seen within the aggregates. The plasma cells have the same heavy and light chain restriction as the B-cells, consistent with a plasmacytoid differentiation of the B-cell clone. The histological pattern does not display features typically found in LPL (39), such as fibrosis, paratrabecular location of lymphoid infiltrates, lymphoplasmacytoid cell morphology, or infiltration by mast cells (5). The lymphoid infiltration mimics that of MZL by morphology; the immune phenotype is not distinct, and some similarities in molecular genetic features have also been identified (5). However, CAD patients do not have an extramedullary marginal zone lymphoma, and therefore, bone marrow involvement of MZL can be ruled out. In summary, CAD-associated LPD does not display the features of other indolent B-cell lymphomas types as described by the WHO classification. Therefore, it should be considered a distinct entity (5, 14, 39, 40). Development of diffuse large B-cell lymphoma is uncommon, probably occurring in less than 4% of the patients during 10 years (18).

Although cold hemagglutination was described in mammals already in 1903 and a CA was discovered in human serum in 1918 (8, 42), the physiological function of CAs has not been clarified. It is difficult to envision a functional role of antibodies with a temperature optimum way below body temperature. Comparative studies, however, have strongly indicated that the evolution of the adaptive immune system began with the jawed vertebrates (43–46). Cartilaginous fish, which are phylogenetically ancient and considered closely related to the first jawed vertebrates, have only one immunoglobulin class in common with humans: IgM (43, 46). The V, D, and J gene sequences, known to undergo rearrangement during B-lymphocyte maturation in humans (47), have also been identified in sharks, however in the form of preformed combinations located on several chromosomes (43). While the *IGHV4-34* gene is not known to produce any physiologically functional antibody in man, the temperature optimum of CAs is much closer to the environmental and body temperature of non-mammal sea vertebrates. Furthermore, CAs can react with antigens other than RBC surface macromolecules, and structures closely related to the I antigen are present on some microorganisms such as *Streptococcus* and *Listeria* species (48, 49). Thus, one might explain human CAs as remnants of a primitive vertebrate immune system (45, 46, 50).

### Immunological Properties of Cold Agglutinins

Most CAs have specificity for the Ii blood group system of carbohydrate antigens (51, 52). The densities of I and i antigens on the RBC surface are inversely proportional. Only the i antigen is expressed on neonatal RBCs, whereas the I antigen predominates from 18 months-age and onwards (51). Hence, in most subjects but infants, CAs specific for the I antigen are more pathogenic than those with anti-i specificity (53, 54). Occasionally, CAs are specific for the RBC surface protein antigen Pr, and these CAs can be highly pathogenic (54, 55). Antigen specificities in CAD and CAS are listed in Table 2. CAs in CAD are usually anti-I specific.

Most CAs in CAD are monoclonal IgMκ (18, 56). Only ~7% of the cases show λ light chain restriction, while CA of the IgG class occurs in less than 5% (18, 57). Monoclonal IgA is even rarer and may not be identical to the CA but rather a bystander (58–60). In CAS secondary to aggressive B-cell lymphoma, the light chain phenotype can be λ as well as κ (61). CAs in infection-associated CAS are polyclonal (2). These CAs are anti-I specific IgM in *Mycoplasma* pneumonia (62, 63) and IgG or IgM with anti-i specificity in EBV or cytomegalovirus infection (25, 64–66). In CAS following infection with EBV, a rheumatoid factor-like IgM-IgG complex has been reported to act as a CA in single case (66).

The activity of a CA is usually assessed by the titer, measured at 4^o^C and defined as the inverse of the maximum serum dilution at which agglutination can be seen. Nearly all patients with CAD have a CA titer > 64; we found a median titer of 512 (range, 16–819200) (3, 13, 18, 67). A titer as high as 168 million has been reported (68). In older publications, titers tended to be higher than reported in more recent literature (9, 11, 18, 21, 69), probably because of an underestimation in clinical practice and some recent studies. Titration can be time-consuming, and some laboratories discontinue the serial dilution when a clearly pathological titer has been reached.

The thermal amplitude (TA) is defined as the highest temperature at which the CA will react with the antigen (9, 70). The pathogenicity of CA depends on the TA more than on the titer (70, 71). If the TA exceeds 28-30^o^C, RBCs will agglutinate in the cooler parts of the circulation even at mild ambient temperatures, often followed by complement activation and hemolysis. In some patients, the TA can approach 37^o^C (53).

For CA detection, serum protein electrophoresis, and assessments of CA titer, TA and IgM levels, samples must be obtained and handled as indicated in Table 4 (10, 13). Detectable CA is present in serum in a proportion of the adult population without any hemolysis or clinical symptoms, although reported percentages are highly variable (53, 72). These individuals do not have CAD or CAS. The normally occurring CAs are present in low titers, in most cases below 64 and nearly always below 256, have low TA, and are polyclonal (53). In contrast, significant CA activity was found in 8.5% of 172 consecutive individuals with a monoclonal IgM (69). Titers were between 512 and 65,500, and all individuals with detectable CA had hemolysis. Therefore, monoclonal CA are generally more pathogenic than polyclonal CA.

**Table 4:** Cold agglutinin disease: handling of samples.

**Analysis**	**Material**	**Sampling**	**Handling of sample**
Hemoglobin, blood cell counts	Blood	EDTA vacutainer	Prewarm at 37–38^o^C before analysis if problems with agglutination
Cold agglutinin titer, thermal amplitude, immunoglobulin quantification, electrophoresis, immune fixation	Serum or plasma	Blood is drawn into prewarmed vacutainers (For serum: No gel or additive). Place in warming cabinet or water bath at 37-38^o^C	Keep at 37–38^o^C until serum/plasma has been removed from the clot/cells, after which the sample can be handled at room temperature
Flow cytometry	Bone marrow aspirate (Too low sensitivity if performed in peripheral blood)	Add EDTA or heparin	Prewarming before analysis will often be sufficient. If not, wash cells at 37-38^o^C. For description, see Ulvestad et al. (9)

*EDTA, ethylenediamine-tetraacetic acid*.

*Table first published in Journal of Blood Medicine 2019 by Berentsen et al. (14), reused under general permission (Creative Commons Attribution Non-Commercial License). Copyright: Berentsen et al*.

**Figure 2:**
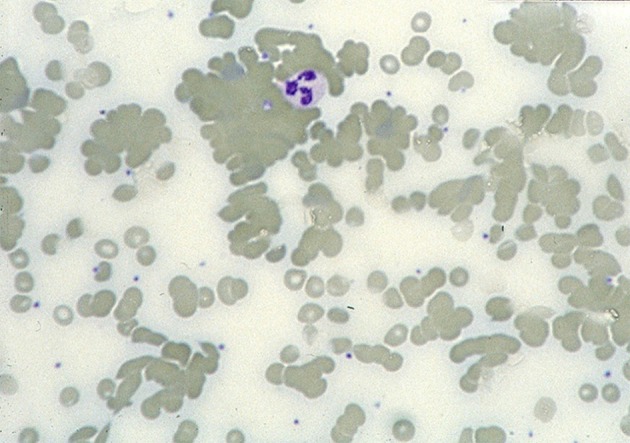
Blood smear in a patient with CAD. Agglutination of erythrocytes dominates the picture. Courtesy of G.E. Tjønnfjord. First published in Clin Adv Hematol Oncol 2020 by S. Berentsen et al. (41), reused under Creative Commons Attribution Non-Commercial License. Copyright: S. Berentsen, A. Malecka, U. Randen, and G.E. Tjønnfjord.

The ability of CAs to agglutinate RBCs after binding to the cell surface (Figure 2) can be attributed to the pentameric structure and large molecule size of IgM (8, 11, 41). Agglutination-mediated, cold-induced ischemic symptoms from the acral capillary circulation have been reported in 40–90% of patients with CAD. Severity can range from slight acrocyanosis to disabling Raynaud phenomena and, in rare cases, even gangrene (16, 18). Atypical circulatory features include dermatologic manifestations, sometimes described as livedo reticularis and sometimes as livedo racemosa (73–75). On rewarming in the central circulation, CA detaches from the cells and the aggregates disintegrate. Complement protein complex C1, which has bound to the AgAb complex before the CA detaches, remains cell-bound and complement activation ensues.

CAs should be distinguished from cryoglobulins (76). Rarely, however, cold-reactive immunoglobulins have been described that exhibited both CA and cryoglobulin properties (68, 76, 77). The Ii antigen system is also present on granulocytes, monocytes, lymphocytes, and thrombocytes (78–80). While aggregation of neutrophils has been observed in rare cases (78, 81), patients with CAD are not known to have an increased frequency of thrombocytopenia (9).

### Complement Activation and Hemolysis

**Figure 3:**
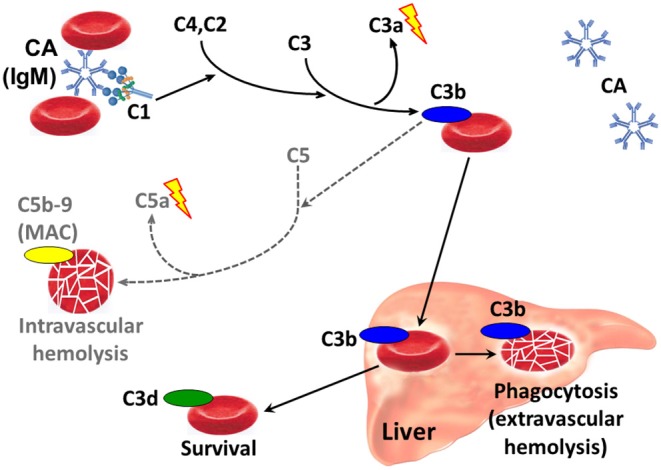
Classical complement pathway-mediated hemolysis in CAD. Only relevant steps and components are shown. Black arrows, major pathways. Gray/dotted arrows, minor pathways. Lightning symbol indicates anaphylotoxin properties. C1, C2, etc., complement proteins; CA, cold agglutinin; Ig, immunoglobulin; MAC, membrane attack complex.

Antigen-bound IgM is a potent complement activator (82–84). Following cold-induced binding of CA to the RBCs during passage through the acral parts of the circulation, the AgAb complex induces fixation of complement protein C1q and, thereby, complement activation by the classical pathway (Figure 3) (84–88). C1 esterase activates C4 and C2, thus generating C3 convertase which results in the formation of C3a, a soluble anaphylotoxin, and C3b, an opsonin with enzymatic activity (84, 89). On rewarming to 37°C in the central circulation and detachment of CA, C3b remains bound and C3b-opsonized RBCs undergo phagocytosis by the mononuclear phagocytic system, mainly in the liver (84, 87, 90, 91). This process is also known as extravascular hemolysis. On the surviving cells, surface-bound C3b is degraded into its more or less inactive split products iC3b, C3c, and C3d.

Complement activation may proceed beyond the C3b formation step by binding of the C4bC2a complex to C3b, thus generating C5 convertase (89, 92). This enzyme initiates the terminal complement cascade by cleaving C5 into C5a, a potent anaphylotoxin, and C5b, which remains cell-bound. C5b is able to bind C6, C7, C8 and C9, resulting in formation of the membrane attack complex (MAC) and intravascular hemolysis. Due to inhibition by surface-bound regulatory proteins such as CD55 and CD59, however, complement activation is often not sufficient to produce clinically significant activation of the terminal complement pathway (73, 87, 93).

The major mechanism of hemolysis in stable disease, therefore, is the extravascular destruction of C3b-coated erythrocytes by the mononuclear phagocytic system. In severe disease and acute exacerbation, however, there can be a substantial component of intravascular hemolysis, as evidenced by the occurrence of hemoglobinuria in 15% of the patients (16), the observation of hemosiderinuria (76), and the modest but significant effect of treatment with eculizumab (73).

IgG is a weaker complement activator than IgM (82, 83), and the rare cases of IgG mediated CAD behave differently from IgM mediated disease in terms of effect of therapy (57). Among the IgG subclasses, IgG3 activates complement more efficiently than does IgG1, whereas IgG2 is a still weaker activator and IgG4 does not trigger the complement system (94). Thus, the mechanisms of hemolysis may be different in IgG mediated disease as compared with typical IgM mediated CAD.

**Figure 4:**
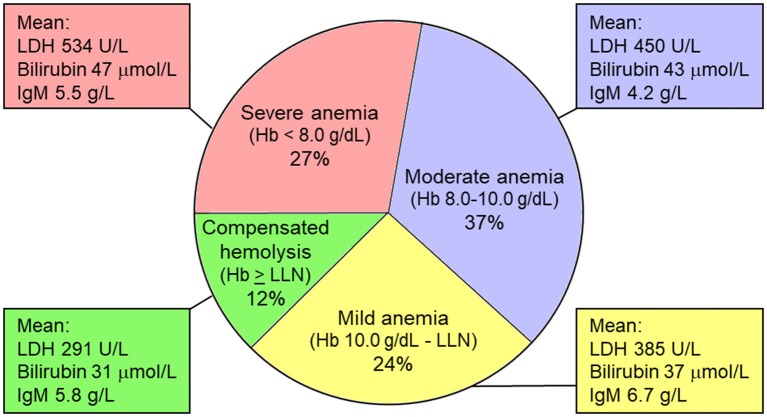
Severity of anemia in cold agglutinin disease (percentages of patients). Severity of anemia correlates nicely with markers of hemolysis (LDH and bilirubin levels), but not with IgM levels, which may be associated with complement-independent RBC agglutinating activity as well as complement-mediated hemolysis. Hb, hemoglobin level; LDH, lactate dehydrogenase; LLN, lower limit of normal (Hb 11.5 g/dL in women and 12.5 g/dL in men). Based on data from Berentsen et al. (18).

In a retrospective cohort of patients with CAD, we found a median hemoglobin level (Hb) of 9.2 g/dL (range, 4.5–15.3 g/dL) (18). Hemolytic anemia was slight (or sometimes even compensated) in 36% of the patients (Hb > 10.0 g/dL), moderate (Hb 8.0–10.0 g/dL) in 37%, and severe (Hb < 8.0 g/dL) in 27%. Severity of anemia correlated with markers of hemolysis, but not with IgM levels, which may be associated with complement-independent RBC agglutinating activity as well (Figure 4).

### Direct Antiglobulin Test

DAT detects immunoglobulin and/or complement components on the RBC surface (3, 95). When markers of hemolysis (lactate dehydrogenase, haptoglobin, bilirubin, and absolute reticulocyte count) show that an anemia is hemolytic, DAT is performed to demonstrate autoimmune pathogenesis. In many laboratories, DAT is first done by using a polyspecific antibody reagent. If AIHA is confirmed, a monospecific DAT must be performed, i.e., by using antibodies against specific immunoglobulin classes and complement proteins. Despite the IgM mediated pathogenesis in CAD and CAS, monospecific DAT is usually negative for IgM because the CA detaches from the RBC surface before it can be identified by DAT (Figure 3). As mentioned above, however, the classical pathway activation and C3b opsonization of RBCs will result in a strongly positive DAT for C3 components, in particular C3d (13, 84). For reasons that are incompletely understood, monospecific DAT is also weakly positive for IgG in up to 20% of the patients (16, 18).

## Cold-antibody AIHA and Inflammation

### Impact of cAIHA on Inflammation

The production of CA by an autonomous B-cell clone explains why CAD does not seem to be associated with other autoimmune diseases (9, 18). Still, serum levels of C-reactive protein (CRP) > 5 mg/L occur in ~25% of the patients in the absence of any identifiable infection, consistent with some kind of proinflammatory state.

Complement activation is closely linked to inflammatory responses and, often, part of these responses (89, 96–99). It has been shown that persistent complement activation is associated with a proinflammatory state in some hemolytic disorders. Interactions between the complement system, inflammatory cytokines, and coagulation was investigated in an *in vitro* model using inhibition of *E. coli*-induced complement activation in human blood (100). The investigators found that complement activation resulted in increased expression of pro-inflammatory cytokines and upregulating of tissue factor mRNA levels. This upregulation, whether induced by *E. coli* or purified lipopolysaccharide, was efficiently blocked by C1 inhibition and, to a lesser extent, by C3 inhibition.

In CAD and CAS, complement activation results in production of C3a, an anaphylotoxin, and, in cases with terminal pathway activation, release of the potent anaphylotoxin C5a (Figure 3) (89, 99). The strong classical pathway activation in CAD is reflected by low serum levels of C4 because of continuous consumption; median level was 0.07 g/L in a large descriptive study (reference range, 0.13–0.32 g/L), and 72% of the patients had levels below 0.13 g/L (18). To a smaller extent, CAD patients also tend to have low levels of C1s, C2, C3, and C5 (18, 87). In a follow-up study of a single patient with CAD, temporarily raised levels of interleukin(IL)-1β, IL-6, tumor necrosis factor(TNF)-α, and interferon(INF)-γ were found to be associated with elevated CRP (101, 102). Other immunoregulatory cytokines may also be involved, as discussed in the next subsection, “Impact of inflammation and infection on cAIHA” (103–105).

In polyclonal autoimmune disorders, release of proinflammatory cytokines have been associated with fatigue, which is a bothersome symptom in many patients with CAD (106, 107). Therapeutic classical pathway inhibition in CAD has been shown to impressively relieve the fatigue, although an effect on fatigue of improved Hb levels cannot be ruled out (107).

In theory, the cross-talk between the complement system, inflammatory processes, and the coagulation cascade (100, 108, 109) might result in an increased frequency of thrombosis in CAD and CAS. Such a risk is definitely present in wAIHA (110, 111). In CAD, this risk has been clearly documented in the most severely affected patients (73), and recent registry-based studies have found a slightly increased frequency of thrombosis in unselected CAD cohorts as well (112, 113). According to one AIHA study, leukocytes influenced the risk of thrombosis (111), and the tissue factor expression by granulocytes has been implicated as one of the links between inflammation and thrombosis (114). The role of this interaction is more unclear in CAD, however, due to the different immune pathogenesis and because CAD patients usually do not have elevated leukocyte counts (9).

### Impact of Inflammation and Infection on cAIHA

Exacerbation of hemolytic anemia during febrile illness in a patient with CAD was described in 1999 as “paradoxical hemolysis” (115). Subsequently, descriptive studies showed that this occurs in 40–70% of the patients (18), and exacerbations have also been described after major trauma or major surgery (101, 116). The phenomenon is best explained by the low levels of classical pathway components, in particular C4, caused by continuous consumption in steady-state patients as already discussed. Probably, C4 activation is rate-limiting for complement-mediated hemolysis because of these low levels. When an acute phase reaction occurs, increased amounts of complement proteins are produced, and exacerbation will ensue. This causal relationship has been documented in a single patient (101). Direct complement activation, for example by microbial agents, may also contribute to the exacerbation in some situations.

In a mouse model of systemic autoimmunity with AIHA as a major component, elimination of the proinflammatory cytokine INF-γ was found to delay AIHA development (102). Moreover, a role of several immunoregulatory cytokines (IL-1α, IL-2, IL-6, IL-10, IL-12, IL-13, IL-17, IL-21, INF-γ, and tumor growth factor(TGF)-β) has been found or implicated in wAIHA (103–105). However, the relevance for CAD of these observations is unclear because of its distinct immune pathogenesis.

In infection-associated CAS, the causal relationship between infection and complement mediated hemolytic anemia is different. In *Mycoplasma pneumoniae* pneumonia, IgM-CA is produced by polyclonal lymphoplasmacytoid cells, probably as part of the physiological immune response (62, 63, 117). In fact, a qualitative test for CA was used as a diagnostic test for *Mycoplasma* infection before specific serology was clinically available, but its usefulness was limited because of low specificity and sensitivity (2). Most of these CAs do not cause hemolysis, but occasionally, profound hemolytic anemia can occur because of high-titer, anti-I specific CAs (62, 63). The temporal course of the disease manifestations is consistent with this explanation, as any hemolytic anemia usually appears rather suddenly in the second week of *Mycoplasma* infection and is self-remitting, usually within 4–6 weeks (2, 63).

## Treatment of CAD

### General Considerations

Non-pharmacological management consists of thermal protection, in particular of exposed parts of the body (3, 7, 11, 118). Some patients even have to avoid cold food and beverages and should not take food from the fridge or freezer without wearing gloves. In my experience, many patients have discovered these precautions before they see the specialist. However, they often need to be explained that these are measures against the hemolytic anemia, not only against the ischemic symptoms. It is equally important for hematologists to provide health care personnel with relevant instructions. In the ward or outpatient department, patients with CAD should keep warm and avoid cold infusions (10, 11, 119). Any bacterial infection should be treated (10, 14, 101). Transfusion, when indicated, can be considered safe. As opposed to wAIHA, in which it is impossible to find compatible donor blood in most cases, compatibility problems are usually not encountered in CAD (2, 118). The patient, including the extremity used for transfusion should be kept warm, however, and most literature recommends the use of an in-line blood warmer (3, 7, 119). Disregarding these precautions has resulted in acute exacerbation, and fatal outcome has been reported (120). Because low complement protein levels are rate-limiting for hemolysis, transfusion of blood products with a high plasma content should probably be avoided (101).

In critical situations where it is not feasible to wait for the effect of specific therapy (see below), plasmapheresis is an option for “first-aid” (121–124). The theoretical rationale for this procedure is strong because virtually all IgM is located intravascularly (124), but no prospective study has been published and some conflicting data do exist (122, 125). Recommendations have been to exchange 1–1.5 times the plasma volume with albumin, not plasma, daily or every second day (7, 123). The effect is short-lived and drug therapy should be initiated concomitantly (7, 10).

According to small retrospective series and case reports, splenectomy has failed to induce remission of CAD (11, 18, 118). This is not surprising, as the extravascular hemolysis mainly takes place in the liver (91). Even though exceptions may exist among the rare patients with CA of the IgG class or with a TA approaching 37^o^C (57, 118), splenectomy should not be used to treat CAD.

The occurrence of CA in subjects who undergo surgery in hypothermia and/or cardiopulmonary bypass remains a challenge. Diverging recommendations exist regarding routine screening for CA in all patients scheduled for such surgery. The frequency of positive findings is low and the consequences of incidentally detected CA not associated with clinical disease have not been clarified (72, 126). Therefore, the cost-effectiveness of such screening is probably low. In patients with CAD, however, a TA measurement and an assessment by a hematologist should be obtained before cardiac surgery, which should be performed under normothermia (116, 126).

Pharmacological therapy for CAD is not indicated in patients with mild anemia or compensated hemolysis in the absence of troublesome clinical symptoms (3, 7, 10). Still, “real life” studies have found that 70–80% of patients have been treated (16, 18). Unlike in wAIHA, therapy with corticosteroids and other unspecific immunosuppression is generally ineffective in CAD (3, 10). Already 30–60 years ago, Schubothe (11) and Nydegger et al. (119) reported the poor efficacy of corticosteroids. Dacie followed 38 patients with “idiopathic” CAD and noticed that only occasional patients had responded to steroids (118). We found a response rate <20% in a retrospective series of 86 patients (18), although some retrospective studies have found a slightly higher percentage of responders (4, 16). Furthermore, in the few patients who do respond, unacceptably high maintenance doses are often required for sustained response (3, 18, 118). Therapy directed against the pathogenic B-cell clone, or, more recently, complement modulation is more likely to succeed (3, 7, 10).

### B-Cell Directed Therapies

Not all B-cell directed treatments have been successful. A small study of chlorambucil therapy found some effect on IgM concentrations and markers of hemolysis, but no significant increase in Hb levels (127). Although IFN-α is not a specific B-cell targeting agent, it has demonstrated favorable activity in a variety of indolent B-cell LPDs (128, 129). However, two small series of IFN-α therapy in CAD showed conflicting results (130, 131). Cladribine monotherapy was found to be ineffective (132). Approximately 25% of patients seem to respond to cyclophosphamide monotherapy.

The first therapy shown to give acceptable response rates was rituximab monotherapy. Two prospective, nonrandomized studies using rituximab 375 mg/m^2^ for four cycles at 1 week interval found partial response (PR) in ~50% of the patients, but complete response (CR) was rare (133, 134). Median response duration was 11 months (range, 2-42 months), and six of 10 retreated patients achieved a second response (133). The treatment was well tolerated. Other authors have reported responses in up to 100% of the patients, but the highest response rates estimated in the literature have been shown to reflect selection and publication bias as well as non-defined or heterogeneous response criteria (135). Although not approved for this indication by EMA or FDA and not available in all countries, rituximab monotherapy has become the most accepted first-line therapy for CAD (3, 7).

Addition of fludarabine was studied in a prospective, nonrandomized trial of 29 patients (136). This regimen (rituximab 375 mg/m^2^ day 1 and fludarabine orally, 40 mg/m^2^ days 1-4 for four cycles at 28 days interval) yielded an overall response rate of 76% (21% CR and 55% PR). Median estimated response duration was 66 months. Grade 4 neutropenia occurred in 14% of the patients, but as much as 59% experienced infection grade 1–3 (136). Based on the use of fludarabine in other indications, there are also some concerns about possible long-term toxicities (137).

Rituximab plus bendamustine combination therapy was prospectively studied in a non-randomized, multicenter trial in which we treated 45 CAD patients with rituximab 375 mg/m^2^ day 1 and bendamustine 90 mg/m^2^ day 1 and 2 for four cycles at 28 days interval (138). This trial had the same inclusion criteria and response definitions as used in the rituximab-fludarabine trial, and the baseline characteristics were almost identical. Thirty-two participants (71%) achieved a response; CR in 18 patients (40%) and PR in 14 (31%). Among 14 patients who had previously received rituximab or fludarabine plus rituximab, response to bendamustine plus rituximab was observed in 7 (50%). Hb levels increased by median 4.4 g/dL in those who achieved CR and 3.9 g/dL in the partial responders. More than 90% of the responders were still in remission after 32 months. Fifty per cent of the responses occurred within 1.9 months, but up to 7 months' time to response was seen in some patients. Neutropenia grade 4 was observed in 9 patients (20%), but only 5 (11%) experienced infection with or without neutropenia. Most clinical adverse events were mild and could be attributed to known non-hematological toxicity of bendamustine.

In a prospective, non-randomized study, 19 patients received one cycle of bortezomib monotherapy. Six participants responded; three responses were graded as CR and three as PR (139). Although these response rates may seem low, the results constitute a promising “proof of principle,” and higher response rates might be achieved by extending the duration of treatment or using bortezomib-based combinations.

In theory, administration of Bruton tyrosine kinase inhibitors or other novel specific B-cell targeted agents would be attractive (140). The rationale for this approach is strong, as studies of WM have showed activity of ibrutinib even in *MYD88* L265P negative cases (141). Our group has successfully treated one CAD patient with ibrutinib, and a systematic study should be done.

We regard four cycles of bendamustine plus rituximab as an efficacious and sufficiently safe regimen that may be considered first-line in relatively fit patients who are severely affected by CAD (3, 10, 138). Safety should be carefully monitored. For other patients who require treatment, rituximab monotherapy should be the first choice (3, 7, 10).

### Complement Modulation

A non-randomized prospective trial that included 12 patents with CAD and one with severe CAS showed some effect of therapy with the anti-C5 monoclonal antibody, eculizumab (73). However, although intravascular hemolysis was significantly inhibited and most patients became transfusion independent, anemia and quality of life scores did not improve significantly. As explained above, C3b-opsonization followed by extravascular hemolysis is the main mechanism of RBC breakdown in steady-state CAD, terminal pathway activation is limited, and C5 is not the optimal target of complement modulation. A meaningful effect has been reported, however, in severely affected patients (142, 143) and as prophylaxis against exacerbation following heart surgery (116), consistent with the notion that intravascular hemolysis may be more prominent in these situations.

Inhibition at the C1 or C3 level would be expected to work better. Plasma-derived C1 esterase inhibitor (C1-INH) is approved for the treatment of hereditary angioedema, which is not a complement-mediated disorder. High doses of C1-INH were shown to block the complement classical pathway, abrogate hemolysis and improve anemia in a patient with a severe, IgM-mediated wAIHA (144), and a similar effect has been reported in a patient with acute, severe CAS (145). However, endogenous C1-INH production is not deficient in AIHA, and further C1-INH therapy would require frequently repeated high doses. C1-INH, therefore, does not seem attractive as long-term therapy, and no systematic trial has been published.

Sutimlimab (BIVV009, TNT009) is a humanized monoclonal inhibitory antibody that targets C1s (146). The murine precursor of sutimlimab, TNT003, showed ability to efficiently inhibit complement activation, C3 deposition, and phagocytosis of RBCs *in vitro* in the presence of normal human serum as a source of complement and patient sera as a source of CA (87). In a phase 1B trial of 10 patients with CAD, weekly intravenous infusions of sutimlimab increased Hb levels by a median of 1.6 g/dL within the first week of treatment and by 3.9 g/dL within 6 weeks (146). According to the results published, “extravascular hemolysis was abrogated in most patients, bilirubin levels mostly normalized within 24 h, and all of six previously transfusion-dependent patients became transfusion-free. Hemolysis recurred after discontinuation, but re-administration of sutimlimab restored the remission” (146). Recently, similar favorable results have been confirmed in a phase 3 trial in transfusion-dependent patients with CAD (107), and a phase 3 trial in transfusion independent patients is ongoing (ClinicalTrials.gov, NCT03347422). Adverse events related to the study drug have not been observed in these trials. Patients received no antimicrobial prophylaxis, but were vaccinated against *Neisseria meningitidis, Streptococcus pneumonia*, and *Haemophilus influenzae* (107, 146).

No clinical results have been published in CAD regarding ANX005, a humanized monoclonal antibody to C1q (147), and peptide inhibitor of C1 (PIC1), a small molecule that targets C1q and blocks the activation of associated serine proteases (148).

Splitting of C3 by C3 convertase is a point of convergence between all three initial complement pathways and critical for activation of the terminal pathway (84, 89). Therefore, inhibition at this level will have the potential to block the entire complement system and is an attractive option in several complement-mediated disorders (149). The C3 inhibitor, pegcetacoplan (APL-2), is a pegylated peptide designed for subcutaneous administration (150). Clinical phase 2 trials have found efficacy of pegcetacoplan in paroxysmal nocturnal hemoglobinuria as well as AIHA (151), and further studies in CAD are warranted. A high risk of infection with encapsulated bacteria might be suspected as a consequence of C3 inhibition, but thus far, clinical data have not supported this concern (150–152). Trial participants were vaccinated as in the C1 modulation trials.

As discussed above, complement-directed therapies for CAD are promising. However, these options are still investigational and will encounter some limitations. First, ischemic symptoms are not complement-mediated and will not be relieved. Second, in contrast to chemoimmunotherapy, treatment will probably have to continue indefinitely to maintain its effect. Third, such therapies will probably be very expensive. On the other hand, the B-cell directed therapies also have obvious limitations: 20–25% of the patients will not respond, the time to response can be several months or even longer (138), and some patients have contraindications or are reluctant to receive treatment with cytotoxic drugs. In contrast, sutimlimab is rapidly acting and seems to have a favorable toxicity profile (107, 146). Undoubtedly, therefore, the upstream complement inhibitors have the potential to fill an unmet need in patients with CAD. It is to be hoped that in severe cases and acute exacerbations, such therapy will “provide a bridge that will allow rapid achievement of remission and transition to B-cell directed treatment when the situation is under control” (88).

## Treatment of CAS

As secondary CAS is even rarer than CAD, no systematic study has been published in this group of disorders, and recommendations have been based on theoretical considerations, case reports, and expert opinion (2, 3, 153). In CAS associated with aggressive lymphoma or other malignancies, no therapy has been established except for treatment of the underlying disease (2, 3, 153).

In infection-associated CAS, optimal antimicrobial therapy should be instituted when relevant (2). Whereas appropriate antibiotic therapy for *Mycoplasma pneumoniae* pneumonia is usually effective for control of the infection, the onset of secondary CAS will often occur after antibiotic therapy has been initiated or even completed (2, 63). This hemolytic anemia is self-remitting but can be profound, and there is an unmet need for therapy in severely affected patients until resolution of hemolysis occurs (63, 154). Corticosteroids have been used in CAS secondary to *Mycoplasma* as well as virus infections (155–157). However, no good evidence of benefit has been published, as all reports are case observations and it is difficult to distinguish between effect of therapy and spontaneous resolution. Transfusions can safely be given provided the same precautions are observed as in CAD.

Temporary use of upstream complement inhibition seems to be an attractive approach based on theoretical considerations, but no clinical evidence has been provided for its effect in secondary CAS. Because of the rarity of this syndrome, prospective trials are unlikely to be performed, but a systematic retrospective study might be feasible on a multinational basis.

## Author Contributions

SB collected the relevant information and references, and wrote the paper.

### Conflict of Interest

Outside this work, the author has received research funding from Mundipharma, lecture honoraria from Apellis, Bioverativ (a Sanofi company), Janssen-Cilag, and True North Therapeutics, and advisory board and consultancy honoraria from Apellis, Bioverativ (a Sanofi company), and Momenta Pharmaceuticals. The reviewer GT declared a past co-authorship with the author to the handling editor
